# Exciton binding energy and hydrogenic Rydberg series in layered ReS_**2**_

**DOI:** 10.1038/s41598-018-37655-8

**Published:** 2019-02-07

**Authors:** J. Jadczak, J. Kutrowska-Girzycka, T. Smoleński, P. Kossacki, Y. S. Huang, L. Bryja

**Affiliations:** 1 Department of Experimental Physics, Wroclaw University of Science and Technology, Wroclaw, Poland; 2 Institute of Experimental Physics, Faculty of Physics, University of Warsaw, Warsaw, Poland; 3 Department of Electronic Engineering, National Taiwan University of Science and Technology, Taipei, 106 Taiwan

## Abstract

Unlike monolayers of transition metal dichalcogenides such as MoS_2,_ which possess high in-plane symmetry, layered ReS_2_ exhibits reduced in-plane crystal symmetry with a distorted 1 T structure. This unique symmetry leads to anisotropic optical properties, very promising for light polarization devices. Here, we report on low temperature polarization-resolved emission and absorption measurements of excitons in ReS_2_ from bulk to monolayer. In photoluminescence and reflectivity contrast spectra we distinguish two strongly polarized excitons X_1_ and X_2_ with dipole vectors along different crystal directions, which persist from bulk down to monolayer. Basing on the PL and RC spectra of bulk crystals we determine the energy of the ground and first four excited states of both excitons, which follow the usual hydrogenic Rydberg series of energy levels of 3D excitonic states (E_n_ = Ry^*^/n^2^). From the numerical fit we estimate that the energy gap is direct and equal to 1671.7 meV and binding energy of X_1_ and X_2_ is equal to 117.5 and 86.6 meV, respectively. In magneto-PL spectra of bulk ReS_2_ up to B = 10 T, the energy shift of all the states is below 2 meV. On reducing the crystal thickness from bulk to monolayer the ground state experience a strong blue shift.

## Introduction

Layered semiconducting transition metal dichalcogenides (TMDCs) have attracted great and current interest, fueled by underlying physics and anticipated applications in optoelectronic and spintronics^1–13^. Similarly to graphene, in monolayers of group VI TMDCs, such as MoS_2_, MoSe_2_, WS_2_, and WSe_2_ the bottom of the conduction band and the top of the valence band are located at the K points of the 2D hexagonal Brillouin zone. On the other hand, contrary to graphene, the lack of inversion symmetry and a strong spin-orbit coupling in single layers of group VI TMDCs results in a valley-contrasting strong spin splitting of the valence and conduction bands which enable excitation of carriers with various combination of valley and spin indices using circularly polarized light^4–7^. However, due to the high symmetry of the crystal structure, their linear optical properties, recorded in absorption and emission spectra, are isotropic in the layer plane. Then again, group VII TMDCs, such as ReS_2_ and ReSe_2_, possesses reduced crystal symmetry compared to the molybdenum and tungsten dichalcogenides which leads to anisotropic in-plane optical properties of ReS_2_ and ReSe_2_^14–17^. Successful preparation of ReS_2_ and ReSe_2_ layers of selected thickness from bulk down to single layer, has given rise to intensive studies of their structural, electronic and optical characterization^18–22^. However, there are many aspects of their physical properties that are still under debate. Tongay *et al*.^18^ have claimed that due to in-plane distortion in ReS_2_ the interlayer coupling is weak and stacked layers in bulk behave as electronically and vibronically decoupled monolayers. In contrast, Aslan *et al*.^19^ in recent photoluminescence (PL) and reflectance spectroscopy experiments on bulk, few- and monolayer ReS_2_ have observed strong linear polarization dependence of the absorption and emission spectra and found that the transition energies of the observed excitons can be tuned with layer thickness. Another controversy is related to the nature of the energy gap of ReS_2_. Some authors have argued that ReS_2_ exhibits a transition from the indirect to direct optical band gap when thinned from bulk to monolayer^20,21^, similarly as in the group VI TMDCs. However in ref.^18^, on the base of photoluminescence and reflectivity measurements, and in recent angle-resolved photoemission spectroscopy (ARPES) experiments^22^, the authors have proven the contrary, i.e. that the energy gap in ReS_2_ is direct independently of the number of layers. Layered character of ReS_2_ suggests a strong enhancement of the Coulomb binding energy of excitons, bound electron-hole pairs (X = e + h) which can lead to their stability even at room temperature. A comprehensive explanation of how excitons are formed is significant both for the comprehension of the underlying physics in such layered materials and for their potential application in optoelectronic devices. Most of optical spectroscopy studies of ReS_2_ in bulk and monolayer form have been performed at room temperature, where absorption and emission lines are broad and overlapping, which hinders detailed analysis of individual excitonic lines.

The aim of this paper is to clarify those issues, and to provide insight into the nature of excitons observed in the optical spectra of ReS_2_ using the accuracy of low temperature experiments. We report on low temperature (7 K) photoluminescence, photoluminescence excitation (PLE) and reflectivity contrast (RC) measurements of ReS_2_ from bulk to monolayer, all of which reveal linearly polarized optical transitions of excitons X_1_ and X_2_. The comparative analysis of PL, PLE and RC spectra allows us to conclude that from bulk down to monolayer the energy gap of ReS_2_ is direct and increases with the decrease of the layer thickness. For bulk crystals we determine the energy of the ground and first four excited states of excitons X_1_ and X_2_ which follow the usual hydrogenic Rydberg series of energy levels of the three dimensional (3D) excitonic states (E_n_ = Ry^*^/n^2^). We establish their large exciton binding energy equal to 118 and 83 meV. We find that both excitons are related to the same energy gap equal to 1671.7 meV. In magneto-PL spectra of bulk ReS_2_ recorded in Voigt configuration up to B = 10 T and temperature *T* = 1.8 K, for both excitons, the energy shift of the ground state is not measurable, whereas the shift for excited states is below 2 meV.

## Results and Discussion

**Figure 1:**
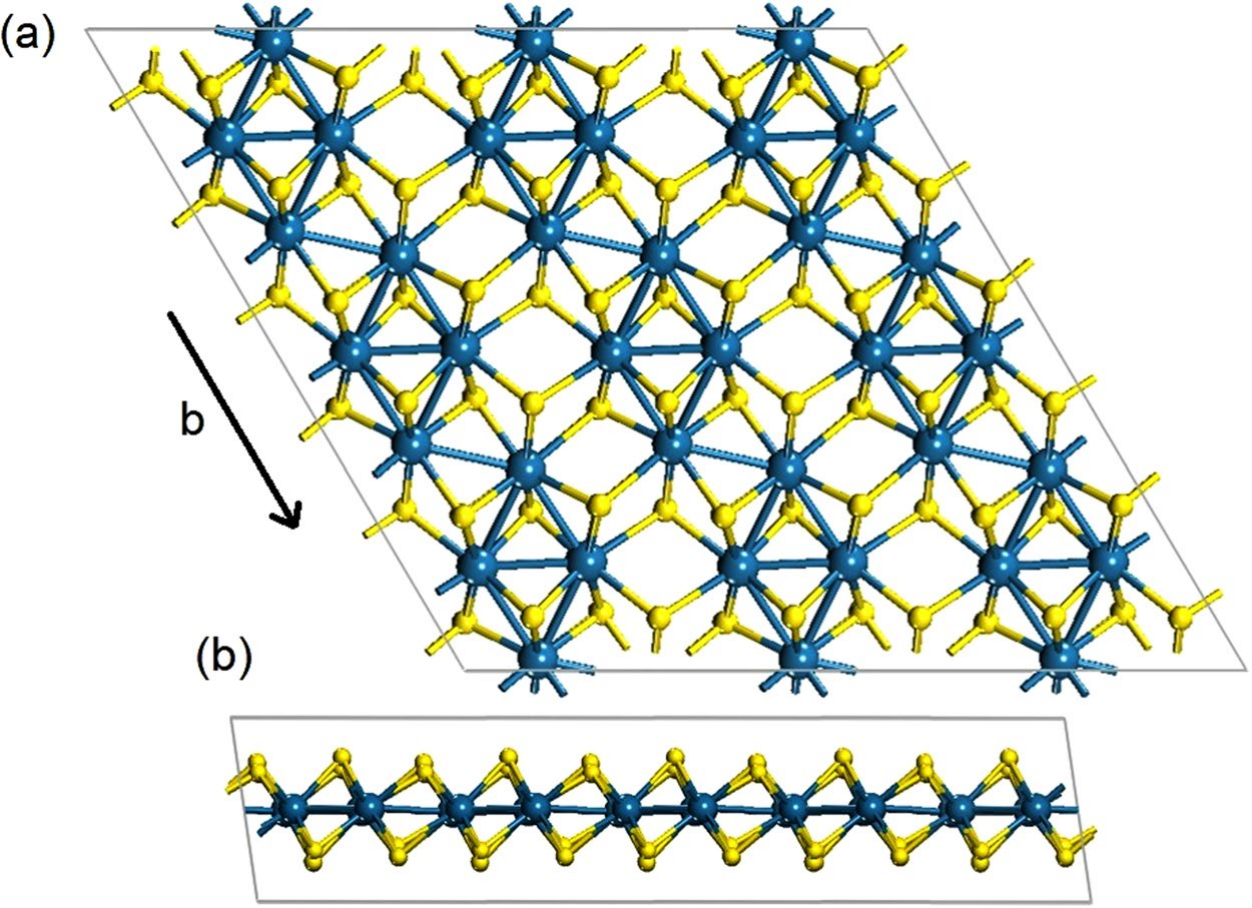
Scheme of monolayer ReS_2_ from: (**a**) top view and (**b**) side view. The Re and S atoms are denoted in grey and yellow, respectively. The Re chain is along the **b** direction.

Layered rhenium disulphide crystallizes in a distorted 1 T diamond-chain structure with triclinic symmetry unit cell^23–25^. Figure 1 presents the crystal structure of a single ReS_2_ layer, from the top and side view, along the **b**-axis. Each layer consists of a sheet of Re atoms located between two S atoms sheets, bound by strong ion-covalent bond between Re and S atoms^22–25^. The S atoms have a distorted octahedral coordination around the Re atoms which results in the formation of Re−Re chains clusters along the **b**-axis^25^. Bulk ReS_2_ crystals are composed of stacks of such layers bound by weak van der Waals forces.

**Figure 2:**
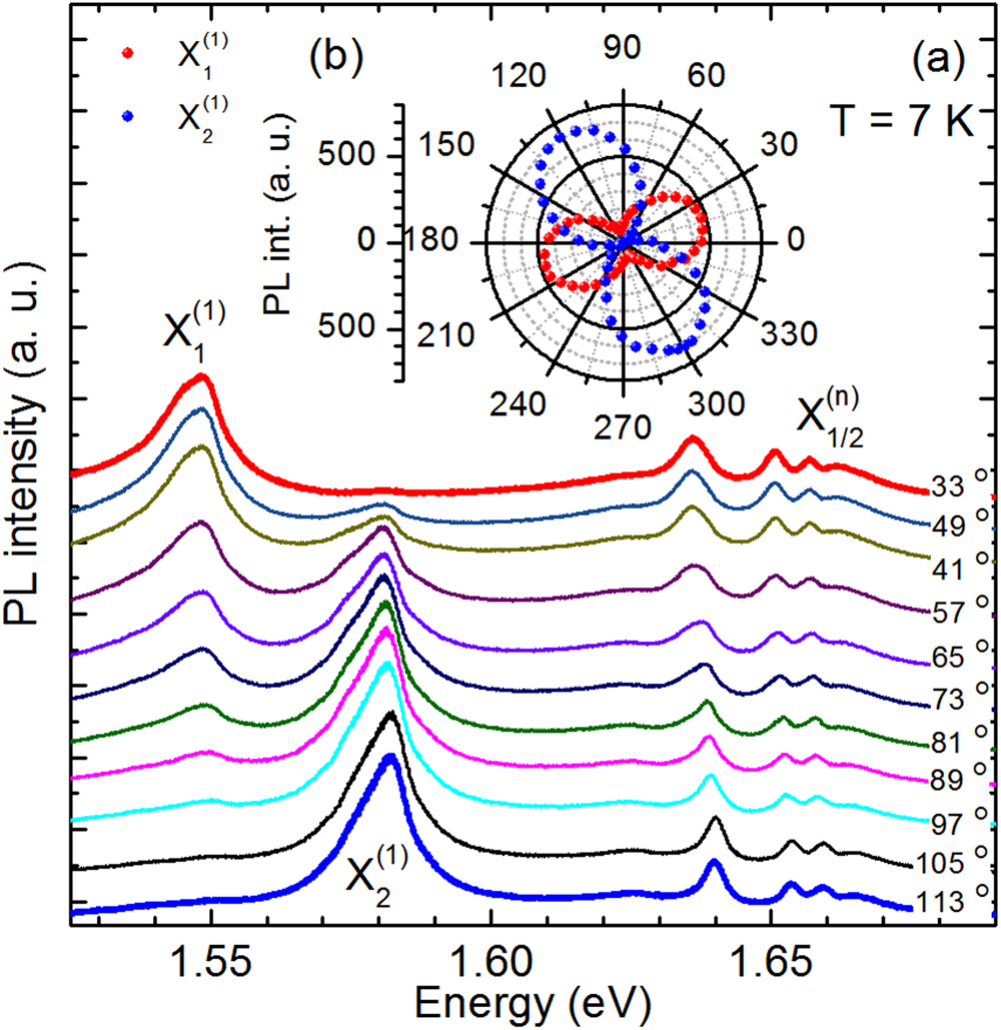
(**a**) Polarization-resolved photoluminescence spectra, measured at 8° intervals from 30° to 120°. (**b**) The integrated PL intensity of the X_1_^(1)^ and X_2_^(1)^ ground states emission plotted as a function of the polarization angle. The data are presented in a polar plot over a 360° range for clarity.

We have studied anisotropic optical properties of ReS_2_ in complementary polarization resolved photoluminescence, photoluminescence excitation and reflectivity contrast measurements. Figure 2 presents polarization-resolved photoluminescence spectra of the bulk ReS_2_ crystal. To omit polarization dependent effects the incident light was circularly polarized and incident photon energy, of 2.33 eV was much higher than the energy of all the observed PL lines. For both the σ^+^ and σ^−^ excitation the PL spectra exhibit the same variation as a function of polarisation, regarding both the shape and energy position of emission lines. Then, the PL spectra were analyzed in linear polarization configuration with the polarization angle of the electric field light (**E**) varied between 30° and 120° with respect to the Re chain axis (**b**-axis) in steps of 4° (for visibility only PL spectra with 8° step are displayed in Fig. 2). For clarity, the spectra are vertically shifted with respect to the 33° measurement. In the low energy sector of the PL spectra we observe two well resolved excitonic peaks which we attribute to the excitonic ground states 1 s and label as X_1_^(1)^ and X_2_^(1)^. Their relative PL intensity changes drastically with the polarization angle but the PL maxima of both lines are detected at the same energy independently of the polarization angle. This implies that these excitons are strongly polarized along different directions of the crystal. In the higher energy sector of the PL spectra we observe four peaks, labelled in analogy to hydrogenic series as 2 s, 3 s, 4 s and 5 s, whose intensity and energy position change as a function of the polarization angle. We tentatively assume that these lines are excited states of the Rydberg series of the excitons X_1_^(1)^ and X_2_^(1)^. According to theoretical predictions optical transitions between the s- like states with zero angular momentum are dipole allowed in most of semiconductors, including semiconducting TMDC^26–28^.

**Figure 3:**
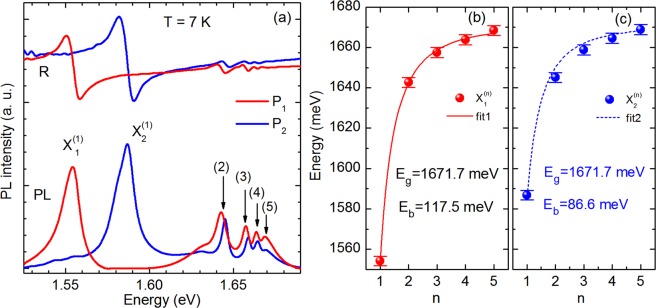
(**a**) Polarization-resolved PL and RC spectra of bulk ReS_2_ recorded in two linear polarisation with the angle between **E** and **b** equal to 33° (red line) and 113° (blue line). (**b**) and (**c**) Experimentally and theoretically obtained transition energies for the 3D exciton states as a function of the quantum number n, for the X_1_ and X_2_ excitons, respectively.

In order to distinguish between the two excitonic series of excited n = 2–5 states we analyse the PL spectra in two opposite polarisations where emission of the X_1_^(1)^ exciton or the X_2_^(1)^ exciton disappears from the spectra. We assume that in the polarization in which X_1_^(1)^ emission is suppressed, the emission from its excited states X_1_^(n)^ (n > 2) is also suppressed and only the hydrogenic Rydberg series of the X_2_^(n)^ exciton is detected. The opposite relation is anticipated for the observation of the X_1_^(n)^ hydrogenic Rydberg series. From the detailed analysis of the evolution of total PL intensity of X_1_^(1)^ and X_2_^(1)^ emission presented in Fig. 2b (the integrated area under the X_1_^(1)^ and X_2_^(1)^ peaks in the ranges from 1.525 eV to 1.562 eV and from 1.562 eV to 1.604 eV, respectively) we find that X_1_^(1)^ related peak disappears at 113° polarization angle, whereas X_2_^(1)^ one at 33°, which is in a good agreement with the recent report of Aslan *et al*.^19^. The PL spectra recorded at these two polarisations are presented in Fig. 3a. The 33° PL spectrum is drawn in red, whereas the 113° PL spectrum is drawn in blue. As it is clearly seen, the peaks of the X_1_^(n)^ excitonic Rydberg series are shifted to lower energies in relation to peaks of the X_2_^(n)^ excitonic Rydberg series. In addition, for both X_1_^(n)^ and X_2_^(n)^ series, we observe the decrease of both the peak intensity and the energy separation for the increasing number of states, which are characteristic features of an excitonic Rydberg series. The peak positions determined as the maxima in PL spectra are plotted in Fig. 3b,c for the excitons X_1_ and X_2_, respectively. To strengthen our interpretation we additionally perform pseudo-absorption reflectivity contrast (RC) measurements. The RC spectra recorded in the same polarization as PL spectra are presented in the upper part of Fig. 3a. In both 33° RC and 113° RC spectra we observe well resolved resonances positioned at the same energies as their counterparts in PL spectra. The observation of the same optical transitions in absorption and emission spectra allows us to conclude that the energy gap in bulk ReS_2_ is direct.

In our estimations of the binding energy of X_1_ and X_2_ excitons we assume that both excitons are related to the same energy gap E_g_, as predicted in recent numerical calculations^29^. Echeverry and Gerber have studied the effect of the interlayer coupling on the band-gap character by means of the self-energy corrected GW method for optimized and experimental sets of structure’s data^29^. They show at the G_0_W_0_ level of theory, when the thickness of the ReS_2_ sample decreases from bulk to bilayer and to a freestanding monolayer, the band gap remains direct at the Γ point of the Brillouine zone, with its energy increasing from 1.6 to 2.0 and 2.4 eV, respectively. In this calculations the valence band maxima and conduction-band minima are double degenerated. This implies up to four excitonic interband transitions with different combinations of spin. The splitting between four excitons is due to the exchange interaction. The observation of four excitons with different optical amplitudes (two strong and two weak) were reported in the optical spectroscopy studies of ReSe_2_ from bulk to monolayer^30^. However, in our studies we observe only two excitons X_1_ and X_2_. In order to calculate the exciton binding energies of X_1_ and X_2_ excitons we compare experimental data with theoretical prediction of hydrogenic Rydberg series. We use a formula typically employed for the description of three dimensional (3D) Wannier excitons in inorganic semiconductors^26^:

1 $${{\rm{E}}}_{{\rm{b}}}^{(n)}={{\rm{E}}}_{{\rm{g}}}-{{\rm{Ry}}}^{\ast }/{{\rm{n}}}^{2}$$

where E_g_ is the energy gap, *n* is the number of exciton state, E_b_^(n)^ is the binding energy at *n*th excitonic state and Ry^*^ is the effective Rydberg constant. The results of a fit are plotted in Fig. 3b,c for X_1_ and X_2_ excitons, respectively. The calculated values of the ground and all excited states are in excellent quantitative agreement with the experimental data for the X_1_ exciton. For the X_2_ exciton the calculated energy of the states 2 s and 3 s are slightly higher than those determined from the experiment. This can be due to the fact that in contrast to the theoretical calculations which predict four different excitonic series^29^ we observe only two.

The observation of two Rydberg series of excitons instead of four can be caused by different optical amplitudes of excitons. Indeed, in the absorption spectra of ReSe_2_ Arora *et al*.^30^ have detected two excitons with high optical amplitudes and two excitons with low optical amplitudes. They distinguished between all four excitons X_1_, X_2_, X_3_ and X_4_ since they are polarized along different directions of the crystal and the weaker features X_1_ and X_3_ are visible for polarizations, where the neighbouring stronger lines, X_2_ and X_4_, respectively are suppressed. In contrast Aslan *et al*.^19^ have reported of the observation of three excitons (exciton 1, exciton 2, exciton 3) in the reflection contrast spectra of ReS_2_ from few-layer to monolayer. However, on the basis of the comparative studies of the evolution of energies and resonance strengths of these three excitons as function of number of layers they have proposed that exciton 3 is an excited (Rydberg) excitonic state of the lower-lying excitons. This scenario is compatible with the increasing separation in energy of exciton 3 from excitons 1 and 2 with decreasing layer thickness. Simultaneously, the thinner samples should exhibit increased exciton binding energy and, hence, increased energy separation between the transitions. This interpretation is consisted with our results and analysis. Namely, we conclude that the broad line named as the exciton 3 in ref.^19^ is indeed related to the superposition of the excited states of the excitons X_1_ and X_2_. In contrast to ref.^19^, in our studies we can resolve between different transition related to the excited states of excitons X_1_ and X_2_ since we investigate bulk ReS_2_ crystals. Nevertheless, as in ref.^19^, we are not able to distinguish between the excited excitonic states in a few-layer ReS_2_.

The energy gap estimated from the Eq. 1 equals 1671.7 meV, which is higher than the result of 1600 meV obtained in recent theoretical calculations^29^. The Coulomb binding energies of excitons X_1_ and X_2_ equal to 117.5 and 86.6 meV are very high in comparison to the exciton binding energy in typical semiconductors belonging to the II-VI and III-V groups, e.g. E_b_ = 4.8 and 10 meV in GaAs and CdTe, respectively^26^. The hydrogenic Rydberg excitonic series in bulk ReS_2_ was previously studied by Ho *et al*.^17^ by means of polarized photoreflectance spectroscopy. However, in contrast to our experiments, they compared photoreflectance spectra with optical polarizations along and perpendicular to the **b** axis. They assumed that the two series of excitons are related to the splitting of the top of the valence band equal to 5 meV. As a consequence they estimated the Coulomb binding energy of the excitons X_1_ and X_2_ equal to 157 and 152 meV. Since the X_1_ and X_2_ excitons are 3D Wannier excitons, we calculate their relative Bohr radius using the well know relations for hydronic like excitons:

2 $${{\rm{E}}}_{{\rm{b}}}/{\rm{Ry}}={{\rm{m}}}_{{\rm{ex}}}/{{\rm{\varepsilon }}}^{2}$$

and

3 $${{\rm{a}}}_{{\rm{ex}}}/{{\rm{a}}}_{{\rm{H}}}={\rm{\varepsilon }}/{{\rm{m}}}_{{\rm{ex}}},$$

where: Ry = 13.6 eV and a_H_ = 0.53 A are Rydberg constant and Bohr radius of the hydrogen atom, respectively; m_ex_ is a relative effective mass of an exciton, ε is the relative dielectric constant of ReS_2_ and a_ex_ is the exciton Bohr radius. We estimate the relative effective masses of X_1_ and X_2_ excitons (1/m_ex_ = 1/m_e_ + 1/m_h_) using the tensor of the electron effective mass obtained from the electrical transport study of multilayer ReS_2_ with polymer electrolyte gating^31^ and the tensor of the hole effective mass obtained in the study of bulk ReS_2_ by means of high resolution angle-resolved photoemission spectroscopy^22^. We obtain effective masses of the X_1_ and X_2_ excitons equal to ~0.33 and ~0.39 (in units of electron mass), respectively, From Eq. 2 we obtain two dielectric constants in directions along and perpendicular to **b** axis equal to ~6.2 and ~7.8 and finally from Eq. 3 we estimate the exciton Bohr radius to be equal ~1 nm for both X_1_ and X_2_ excitons.

**Figure 4:**
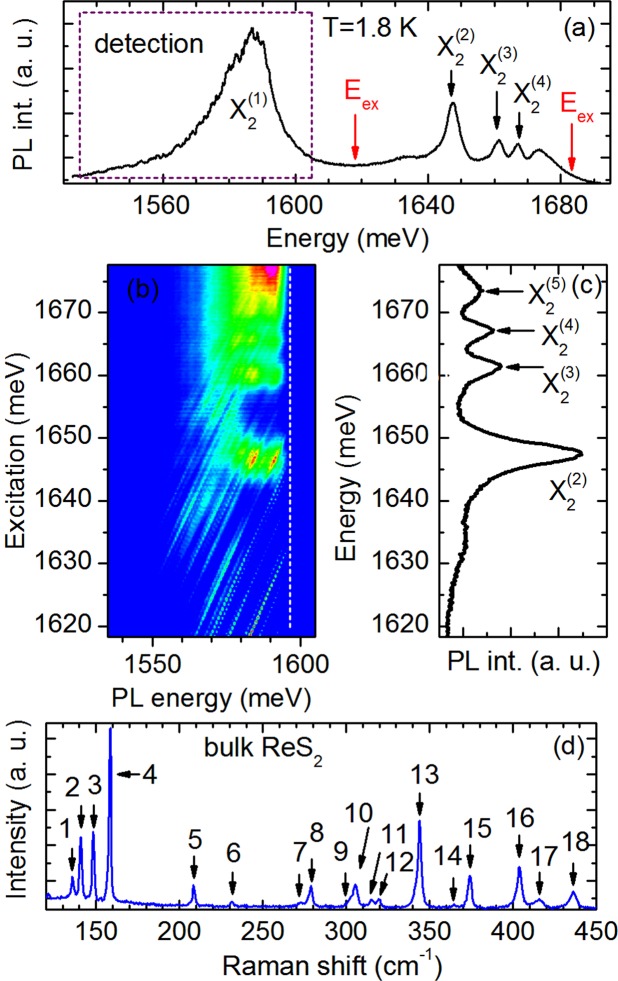
The photoluminescence and photoluminescence excitation spectra of bulk ReS_2_ recorded at *T* = 1.8 K. (**a**) The 113° PL spectrum. The energy regions of detection and excitation of PLE signal are marked by dotted lines and arrows, respectively. (**b**) The 113° PLE signal plotted as a color map. Red and blue color indicate high and low intensity, respectively. (**c**) The 113° PL spectrum presented in the energy region of PLE signal.

To gain further insight into the nature of the excitons X_1_ and X_2_ we have performed additional measurements of photoluminescence excitation and magneto-photoluminescence of bulk ReS_2_. Figure 4 presents polarised 113° PL and 113° PLE spectra in which only the Rydberg series of the exciton X_2_ is detected. The PLE signal is presented in Fig. 4b as a color map. The blue color is related to low signal whereas the red color is related to high signal To facilitate the analysis of the PLE signal the PL spectra in relevant energy regions are displayed in Fig. 4a,c. The PLE signal was detected in the energy range related to the ground exciton state from 1535 to 1605 meV. The laser excitation energy was tuned across the energy of the excited states 2 s, 3 s, 4 s and 5 s from 1619 to 1676 meV. The features observed in the low energy sector of 113° PLE spectra are related to Raman active optical phonons. For comparison, Fig. 4d shows the non-resonant (2.33 eV), unpolarized Raman scattering spectra of bulk ReS_2_. In the 120–450 cm^−1^ range we can distinguish up to 18 phonon modes (marked by arrows) whose energies agree well with the previous studies of Raman spectra of ReS_2_^32^. As it is seen from the comparison of PLE and PL spectra in Fig. 4b,c the energy position of all the related peaks are detected at almost the same energies which confirms our interpretation of these lines as Rydberg series of the exciton X_2_. The same relations are observed between 33° PL and 33° PLE spectra in which only the Rydberg series of the exciton X_1_ is observed.

**Figure 5:**
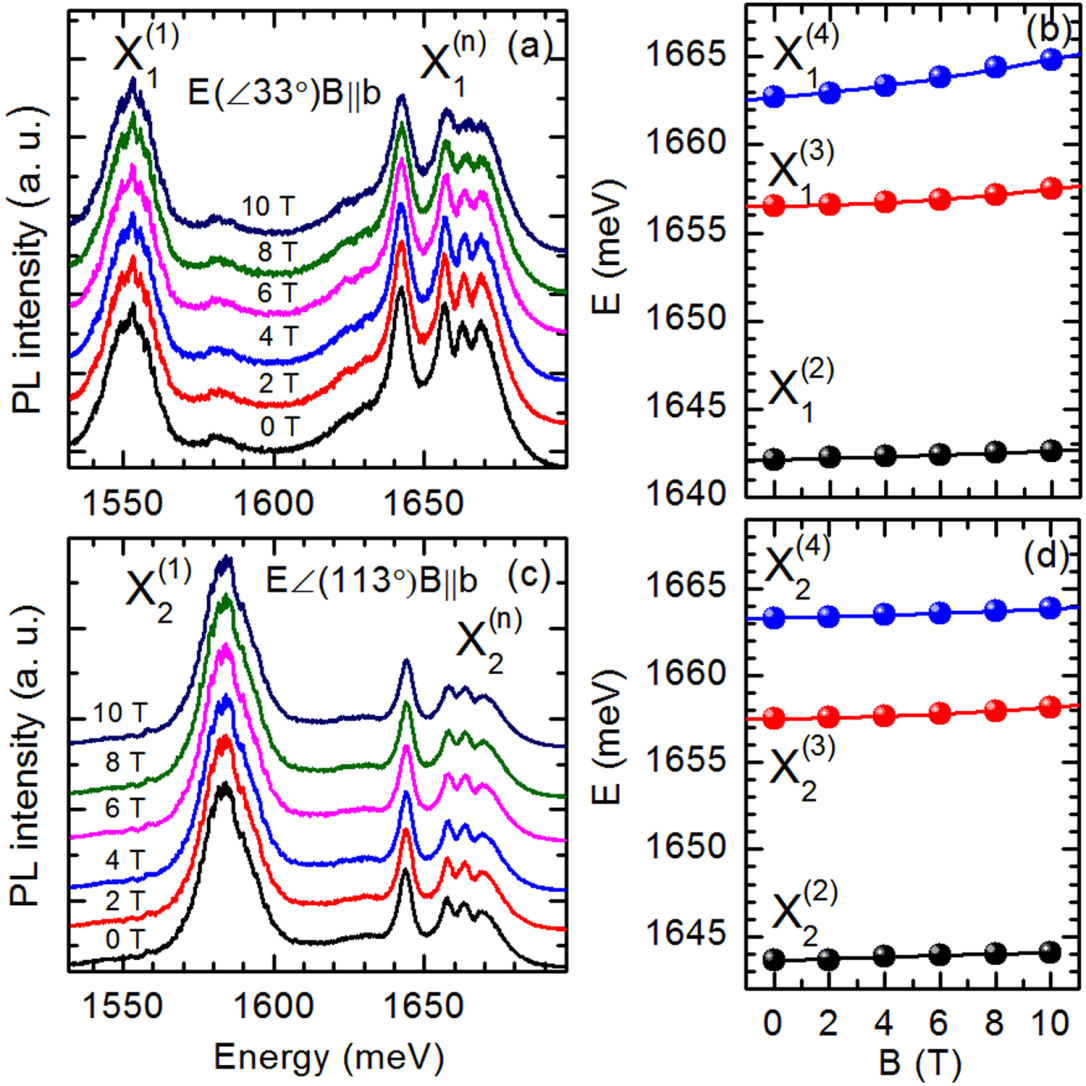
(**a**) and (**c**) Magnetic field evolution of PL spectra of bulk ReS_2_ at T = 1.8 K in polarizations E(<33°) B||b and E(<113°) B||b, respectively. (**b**) and (**d**) Energies of different excitonic transitions extracted from PL spectra recorded in σ and π polarizations, respectively.

The magneto-photoluminescence measurements have been conducted in the Voigt configuration with magnetic field up to 10 T with a 1 T step applied along the **b** axis (**B**||**b**)^33^. Due to the strong anisotropy of the in-plane optical properties resulting from triclinic symmetry of bulk ReS_2_ we have not performed experiments in Faraday configuration with magnetic field perpendicular to the plane through b axis (**B**⊥**b**). However, the Faraday configuration is commonly used in the magneto-spectroscopy studies of the exciton diamagnetic shifts in the high symmetry structures, for example, in the monolayer group-VI transition metal dichalcogenides^12^ or in the two dimensional GaAs/Ga_1−x_Al_x_As structures^34–36^. In Fig. 5a and c the PL spectra recorded in the magnetic fields from 0 to 10 T with a 2 T step are presented for two distinctive linear polarizations (E(33°)B||b) and (E(113°)B||b) (as in Fig. 3a), respectively. The magnetic shift of all the observed lines is very small. For the ground states of the excitons X_1_ and X_2_ the shift is not measurable, whereas for the excited states it is less than 2 meV for the highest applied magnetic field B = 10 T. This observation mainly confirms the strong Coulomb binding of the both excitons. Due to the large width of emission lines (full widths at half maximum (FWHM) equal to ~15 meV and ~7 meV for the ground and excited states of X_1_ and X_2_ excitons, respectively) the Zeeman splitting is not observed and the accurate estimation of the Lande g factor and diamagnetic constant a_n_ from the formula:

$${{{\rm{\Delta }}E}^{({\rm{n}})}}_{{\rm{B}}}=\pm \,1/2\,{g{\rm{\mu }}}_{{\rm{B}}}{\rm{B}}+{{\rm{a}}}_{{\rm{n}}}\,{{\rm{B}}}^{2}$$

used for low magnetic field limit^33,37^ is not possible. More detailed verification of the influence of the magnetic field on the ground and excited states in ReS_2_ crystals would necessitate further measurements at higher magnetic field and theoretical calculations of the diamagnetic coefficient of an exciton in layered semiconductor structures.

**Figure 6:**
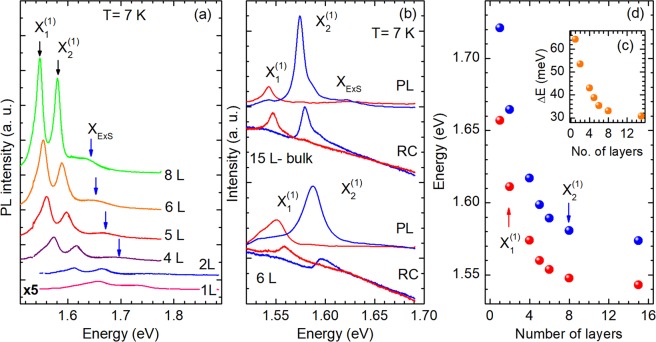
(**a**) Unpolarized PL spectra of 1 L, 2 L, 4 L, 5 L, 6 L and 8 L ReS_2_ flakes. (**b**) Polarization-resolved PL and RC spectra of 15 L and 6 L flakes recorded in two linear polarization with the angle between **E** and **b** equal to 33° (red line) and 113° (blue line). (**c**) Energies of different excitonic transitions extracted from PL measurements.

Finally, we perform optical spectroscopy measurements of ReS_2_ flakes. Figure 6a presents unpolarized PL spectra of ReS_2_ flakes with different number of layers. The Supplementary Fig. 1 presents optical microscope image of the studied ReS_2_ flakes prepared by mechanical exfoliation and deposited on target SiO_2_(295 nm)/Si substrate. The number of layers have been tentatively estimated by optical contrast and then the thickness of the particular areas of ReS_2_ structure have been determined by means of AFM measurements, presented in Supplementary Figs 2a–2s). For all the studied flakes in the low energy sector of PL spectra two emission lines are detected, which by comparison to PL spectra of bulk crystals we have attributed to optical transitions of the ground states of excitons X_1_ and X_2_. However, in contrast to bulk ReS_2_ crystals, we are not able to resolve between the excited states of excitons X_1_ and X_2_ in the PL or RC spectra. We observe only the broad PL feature at the higher energy (marked as ExS in Fig. 6a). Moreover, as in the bulk ReS_2_ crystal, the excitons X_1_ and X_2_ are strongly polarized in emission and pseudo-absorption spectra. In Fig. 6b the polarized PL and RC spectra for 15 and 6 layers samples are presented (see also Supplementary Fig. 3 in Supplementary Materials). The total PL intensity of both excitons strongly increases with the number of layers, and starts to saturate for flakes thicker than 8 layers, which is seen in Fig. 6a. The similar increase of PL intensity of ReS_2_ with the increased number of layers have been recently reported at room temperature experiments^18^. This is in contrast to the behavior observed in the group VI TMDCs, such as MoS_2_, MoSe_2_, WS_2_ and WSe_2_, where the PL intensity of the monolayer increases by orders of magnitude due to the crossover from an indirect band gap in the bulk to a direct band gap in the monolayer. According to the recent theoretical calculations of Echeverry and Gerber^29^ in layered ReS_2_ the direct band gap occurs at the Γ-point of the Brillouin zone irrespective of the crystal thickness. Hence, the oscillator strength of excitonic transitions should increase simultaneously for thicker samples. Moreover, with decrease of the number of layers, from 15 L to 1 L (Fig. 6d), both X_1_ and X_2_ excitons exhibit strong blue shift, which is 114 meV and 146 meV, respectively. Also, their relative energy separation increases, as shown in Fig. 6c. These large shifts are in strong contrast to other, well known group VI TMDCs, where the shifts for the ground state A excitons are much smaller and are in the order of tens meV^38,39^. It is likely due to the stronger electron-hole coupling at Γ-point than at K-point, where direct band gap occurs for the monolayer MoS_2_, MeS_2_, WS_2_ and WSe_2_. In the case of group VI TMDCs, an increasing band gap with decreasing flake thickness compensates the effect of an increasing excitonic binding energy^40^ resulting in a weak dependence of exciton transition energy on the layer thickness. In ReS_2_, observed increase of the exciton transition energy caused by quantum confinement is less compensated. Moreover, this energy change is different for excitons X_1_ and X_2_ likely due to induced in-plane anisotropy.

## Conclusion

Summarizing, we have studied at low temperature the polarization-resolved emission and absorption of excitonic states in layered ReS_2_, from bulk to monolayer. In photoluminescence, photoluminescence excitation and reflectivity contrast spectra we distinguish two strongly polarized excitons X_1_ and X_2,_ with dipole vectors along different crystal directions, which persist from bulk down to monolayer. The detailed analysis of optical spectra allows us to conclude that in ReS_2_ from bulk to monolayer the energy gap is direct. We have experimentally determined the energies of the ground and first four excited excitonic states of the fundamental optical transition in bulk ReS_2_ for excitons X_1_ and X_2_ and found that they follow the Rydberg series of energy levels of the three dimensional excitonic states. From the numerical fit we have calculated the energy gap of bulk ReS_2_ equal to 1671.7 meV and the binding energy of excitons X_1_ and X_2_ equal to 117.5 and 86.6 meV, respectively. In magneto-photoluminescence spectra of bulk ReS_2_ performed in the fields up to 10 T we have detected a very small shift of all the observed peaks, below 2 meV, which confirm strong Coulomb binding of excitons. In optical spectra of the few and monolayer ReS_2_ we can distinguish only the ground states of X_1_ and X_2_ excitons which experience a strong blue shift on reducing the crystal thickness from bulk to monolayer.

## Methods

The studied ReS_2_ crystals were grown by chemical vapour transport technique (CVT). Prior to the crystal growth, the powdered compounds of the series were prepared from the elements (Re: 99.99%; S: 99.999%) by reaction at *T* = 1000 °C for 10 days in evacuated quartz ampoules. The chemical transport was achieved with Br_2_ as a transport agent in the amount of about 5 mg/cm^3^. The ReS_2_ crystals formed thin silver-colored, graphite-like platelets up to 2 cm^2^ in area. The X-ray diffraction patterns confirmed the triclinic symmetry of ReS_2_ with all the parameters consistent with those previously reported^16,23,24^. Hall effect measurements reveal n-type semiconducting behavior. The flakes of ReS_2_ of thickness from bulk down to monolayers were prepared by mechanical exfoliation of bulk crystals. Initially, flakes are exfoliated onto a DGL film (Gel-Pak) attached to a glass plate and identified by their optical contrast and characterized by Raman scattering and PL measurements at 295 K. For further optical studies, they are deposited on the same Si/SiO_2_ (295 nm) target substrate. The number of layers is determined by means of Atomic Force Microscopy measurements. We have measured the thickness of a single ReS_2_ layer to be equal to ~0.7 nm.

The experiments were conducted using two set-ups. In experiments where magnetic field was not applied the samples were mounted on a cold-finger of non-vibrating closed cycle cryostat, where temperature can be varied from 7 to 300 K. Photoluminescence was excited by the 532 nm (2.33 meV) line of a Diode-Pumped Solid State laser. The laser beam was focused on the sample under normal incidence using a 50x high resolution, long distance microscope objective (NA = 0.65). The diameter of the excitation spot was equal to ~1.5 μm. The PL signal was collected by the same objective. The Raman scattering measurements were performed in backscattering geometry. The spectra were analyzed with a 0.5 m focal length spectrometer and a 600 lines/mm grating. A Peltier-cooled Si charge couple device was used as a detector. The reflectivity contrast measurements were conducted in the same set-up, with a filament lamp as a light source. The magneto-optical measurements were conducted in the similar optical set-up, with the use of a bath liquid helium cryostat with a superconducting magnetic coil. The experiments were done in Voigt configuration in magnetic field up to B = 10 T and temperature *T* = 1.8 K.

## Supplementary information

Supplementary Information, Exciton binding energy and hydrogenic Rydberg series in layered ReS2
