# Supplementary material for: Exciton binding energy and hydrogenic Rydberg series in layered ReS2
Source: Sci Rep. 2019 Feb 7;9:1578. doi: 10.1038/s41598-018-37655-8 (PMC6367321; doi:10.1038/s41598-018-37655-8)
Supplement: Supplementary file 1 — Supplementary Information, Exciton binding energy and hydrogenic Rydberg series in layered ReS2 [file 41598_2018_37655_MOESM1_ESM.doc]

**Supplementary Information**

**Exciton binding energy and hydrogenic Rydberg series in layered ReS_2_**

J. Jadczak^1*^, J. Kutrowska-Girzycka^1^, T. Smoleński^2^, P. Kossacki^2^, Y. S. Huang^3^, and L. Bryja^1^

*^1^Department of Experimental Physics, Wroclaw University of Science and Technology, Wroclaw, Poland*

*^2^Institute of Experimental Physics, Faculty of Physics, University of Warsaw, Poland*

*^3^Department of Electronic Engineering, National Taiwan University of Science and Technology, Taipei 106,Taiwan*

**Corresponding author: Joanna Jadczak (joanna.jadczak@pwr.edu.pl)*


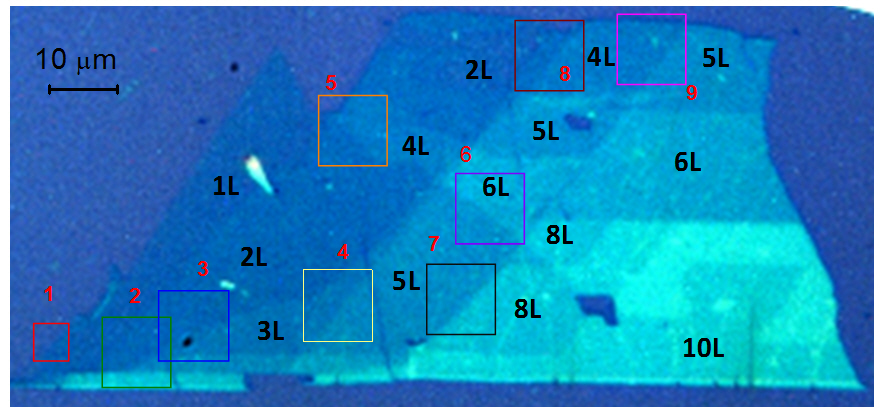


**Supplementary Figure 1** The optical image of a few-layer ReS_2_ sample (from monolayer to 10 layers) deposited on SiO_2_/Si substrate. The colored rectangles indicated by red numbers (1-9) show particular areas of ReS_2_ structure, which are characterized additionally by the AFM measurements (Supplementary Figure 2) and comparative PL and RC measurements (Supplementary Figure 3).

**Supplementary Note 1. Optical microscope image.**

We have investigated two sets of samples. First, the bulk ReS_2_ crystals (thickness of about 100 mm) for polarization-resolved photoluminescence (PL), excitation photoluminescence (PLE), reflectance measurements (RC) and magneto-photoluminescence were directly mounted on a holder in the cryostat. Since the b-axis is parallel to the metal Re cluster chains, which corresponds to the longest edge of the plate ^14^, the configuration for all the experiments is arranged in such way that the longest edge of the crystal is parallel to the edge of the holder. Second, two dimensional layered ReS_2_ structures with varying layer thickness from 1L up to ~20 L for comparative PL/RC measurements were mechanically exfoliated and deposited on target SiO_2_/Si substrate.

The Supplementary Fig. 1 presents optical microscope image of the ReS_2_ flakes prepared by mechanical exfoliation and deposited on target SiO_2_(295 nm)/Si substrate. The number of layers have been tentatively estimated by optical contrast and then the thickness of the particular areas of ReS_2_ structure have been determined by means of AFM measurements, presented in Supplementary Fig. 2.


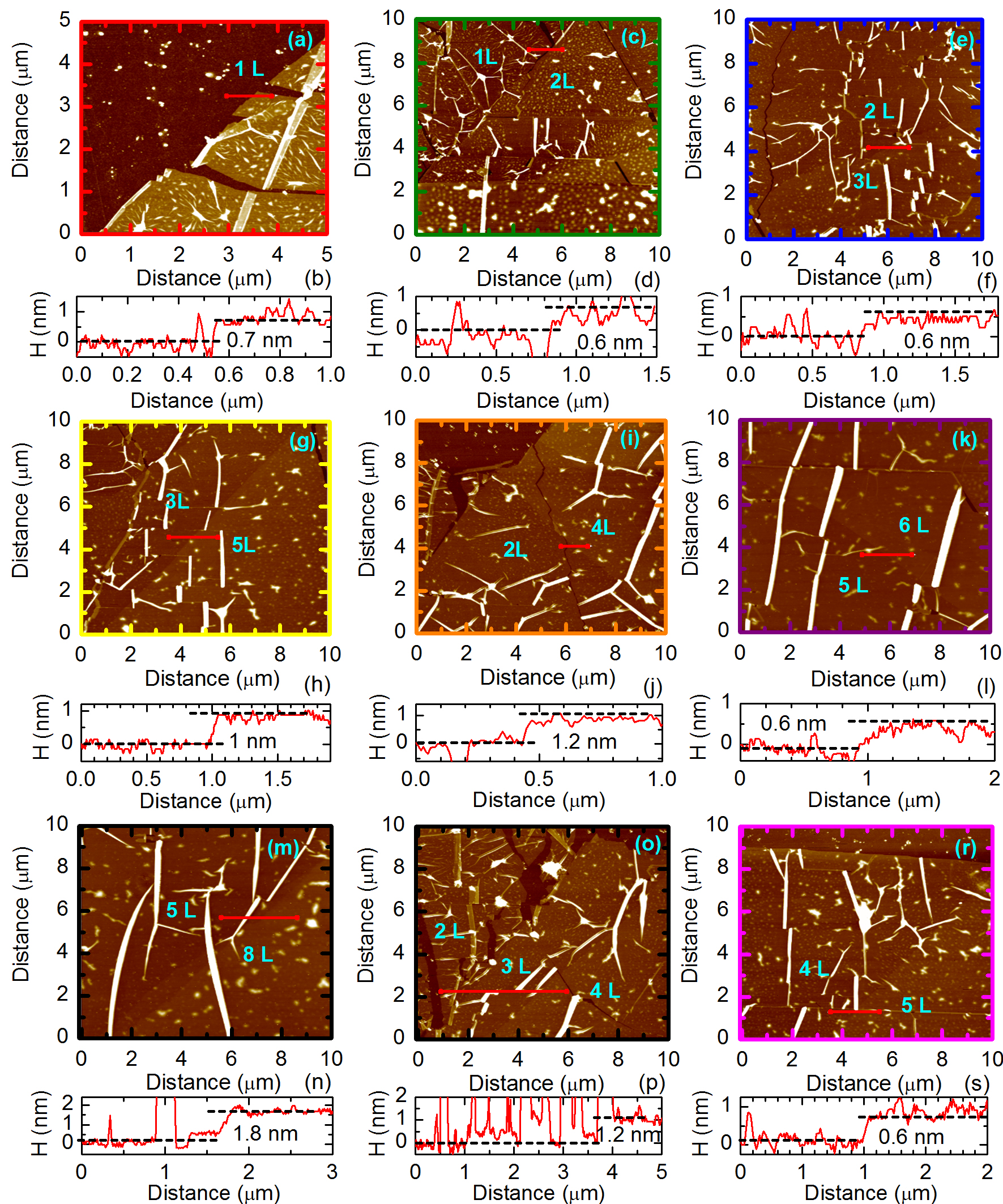


**Supplementary Figure 2** The AFM topography (**a, c, e, g, i, k, m, o, r**) and height profile along the red line (**b, d, f, h, j, l, n, p, s**) of the chosen areas of ReS_2_ layered structure which is presented in Supplementary Fig. 1. The area of scan for monolayer in fig.2**(a)** is 5 by 5 μm, and the monolayer height is ~ 0.7 nm. The area of scan for the rest of the ReS_2_ flakes is 10 by 10 μm.

**Supplementary Note 2. Atomic Force Microscope image.**

Supplementary Fig. 2 (a)-(s) presents the AFM images and height profiles corresponding to the areas indicated by the rectangles in Supplementary Fig. 1s. The thickness of a single ReS_2_ layer measured relative to SiO_2_ surface is equal to ~0.7 nm (Fig. S2 a, b). The thickness step between 1 L and 2 L typically yielded by AFM scan is about ~0.6 nm or 0.7 nm. The thickness discrepancy in Angstrom limit is likely attributed to the instrument offset due to tip-substrate interaction as well as adsorbed molecules (H_2_O) between the monolayer and the SiO_2_ substrate.


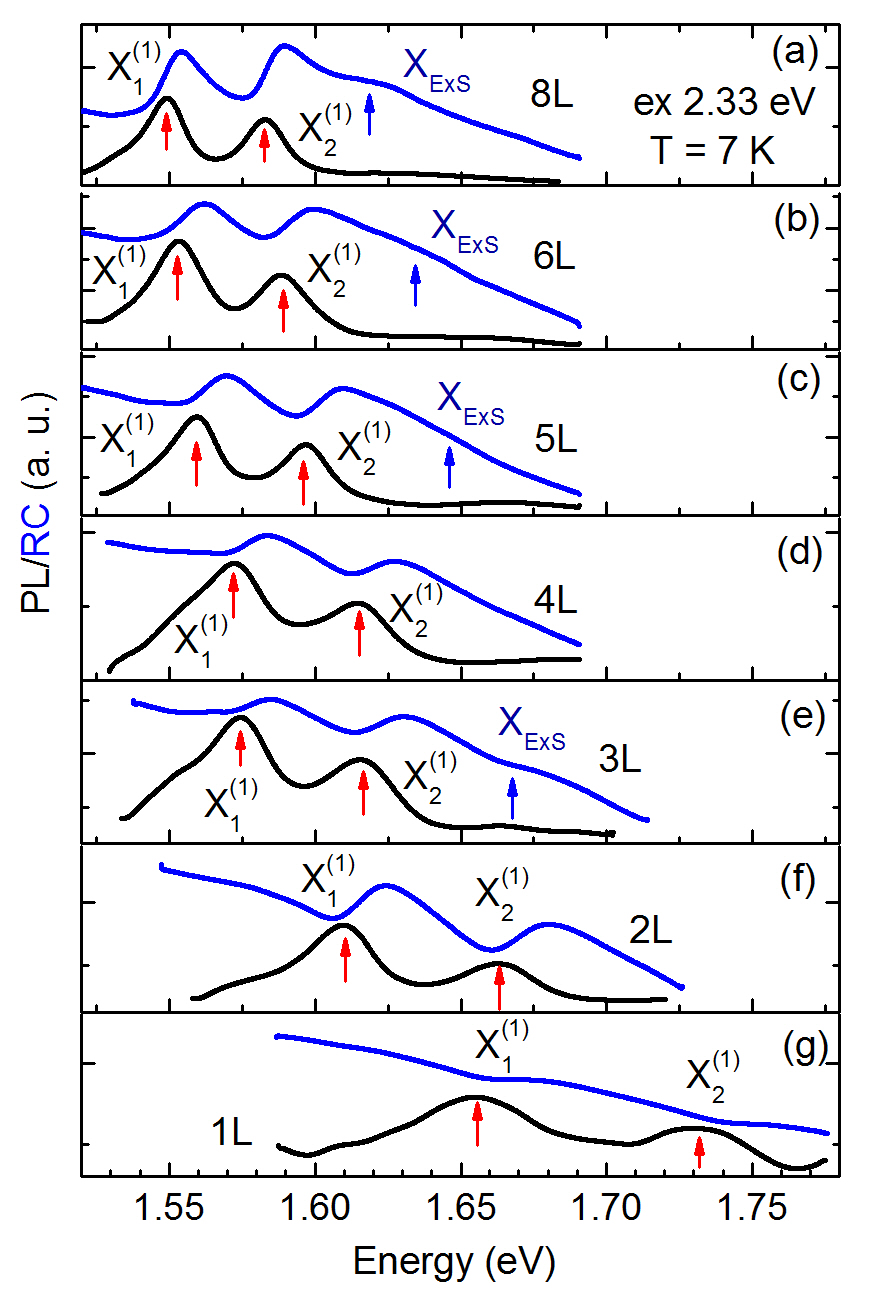


**Supplementary Figure 3** The low temperature (7 K), comparative PL and RC spectra measured in different points of the ReS_2_ layered structure (Supplementary Fig.1), which correspond to decreasing number of layers, from 8 L to 1 L.

**Supplementary Note 3. Comparative PL/RC spectra of layered ReS_2_.**

Supplementary Fig. 3 shows comparative, low temperature (7 K) PL and RC spectra measured in different points of ReS_2_ structure, which correspond to the areas marked in Supplementary Fig. 1.
